# Supplementary material for: Community-based prevalence of rheumatic heart disease in rural Ethiopia: Five-year follow-up
Source: PLoS Negl Trop Dis. 2021 Oct 13;15(10):e0009830. doi: 10.1371/journal.pntd.0009830 (PMC8513824; doi:10.1371/journal.pntd.0009830)
Supplement: S1 Table — (DOCX) [file pntd.0009830.s001.docx]

S1_Table: Age/sex and parental education/occupation in subjects whose cardiac lesions regressed, were unchanged or progressed during the five years of the study.

|  |  |  |  | Parental | |
| --- | --- | --- | --- | --- | --- |
|  | No. | M/F | Mean age (sd) | Education* | Occupation+ |
| Regression | 13 | 6/7 | 17.1 (4.4) | 9 | 8 |
| Unchanged | 20 | 10/10 | 18.1 (3.7) | 6 | 12 |
| Progression | 8 | 5/3 | 16.9 (3.9) | 3 | 5 |

*Less than elementary education, +Farmer/Labourer
