# Supplementary material for: Community-based prevalence of rheumatic heart disease in rural Ethiopia: Five-year follow-up
Source: PLoS Negl Trop Dis. 2021 Oct 13;15(10):e0009830. doi: 10.1371/journal.pntd.0009830 (PMC8513824; doi:10.1371/journal.pntd.0009830)
Supplement: Attachment — Submitted filename: Response_to_reviewers 160821.docx [file pntd.0009830.s002.docx]

Dear Dr Beaton, Dear Dr Zuhlke,

We are grateful to the editors and reviewers for the constructive comments which we have now addressed in fully as detailed in the accompanying letter.

We believe that the manuscript has been significantly enhanced and hope that it is now acceptable for publication in PLOS Neglected Diseases.

We have made major revisions in the manuscript, in order to clarify the aims and objectives of the study and highlight the results and findings.

Please find below our response to the reviewer’s (blue).

With our very best wishes,

Susy

**Reviewer's Responses to Questions**

Key Review Criteria Required for Acceptance? As you describe the new analyses required for acceptance, please consider the following:

**Methods**

-Are the objectives of the study clearly articulated with a clear testable hypothesis stated? -Is the study design appropriate to address the stated objectives? -Is the population clearly described and appropriate for the hypothesis being tested? -Is the sample size sufficient to ensure adequate power to address the hypothesis being tested? -Were correct statistical analysis used to support conclusions? -Are there concerns about ethical or regulatory requirements being met?

Reviewer #1: No

As suggested by the reviewer we have rewritten the introduction and clarified the objectives of the study.

Reviewer #2: Yes. Objectives, study design are well described. Sample volume was not applicable since it is a follow up study of RHD patients.

Reviewer #3: The authors present 5-year follow-up data on patients with subclinical RHD from Ethiopia. Theycompare progression and regression rates with a similar number of controls. It would be useful for the authors to provide details on how they chose the controls. Were they chosen randomly? What was the sampling population? Was any matching performed? What was the rationale for choosing the number of subjects that they did?

Controls were matched for age and gender. To do this we used the original survey register to systematically identify the next 1-2 subjects of the same gender as the case and within two years of age. All controls had been screened during the original study and did not have any features of RHD. (details now provided on page 3 Line 62)

The sample size of the original study was chosen to estimate the prevalence in this population with a standard error of 1.0%.

--------------------

**Results**

-Does the analysis presented match the analysis plan? -Are the results clearly and completely presented? -Are the figures (Tables, Images) of sufficient quality for clarity?

Reviewer #1: No

Reviewer #2: Results are described in detail. Authors should also mention the number of cases that were clinically diagnosed based on auscultation initially.

At the time of the initial study, all subjects were asymptomatic and did not have clinical signs of RHD.

Reviewer #3: The reported estimates of progression and regression are consistent with the published literature. For the subjects who showed progression, readers may wish to know more details regarding how the disease progressed. What were the lesions? How many valves were affected? Were the lesions moderate or severe?

Of the 23 subjects with definite RHD at follow-up, 17 had mitral valve disease, three had aortic valve disease and three mixed valve disease (page 6 line 114).

As this was a survey carried out by a local cardiologist in difficult circumstances, for simplicity we restricted the classification of RHD to the current WHF criteria (as shown in Table 1) and did not attempt obtain more detailed data on the nature of the lesions.

--------------------

**Conclusions**

-Are the conclusions supported by the data presented? -Are the limitations of analysis clearly described? -Do the authors discuss how these data can be helpful to advance our understanding of the topic under study? -Is public health relevance addressed?

Reviewer #1: No

Reviewer #2: Conclusions are supported by the data. Limitations described but not in detail. The loss of follow up could affect the results of the study, since the numbers are significant.

We have re-written the discussion to try and answer these points and in particular the limitations of a relatively small study with significant loss to follow-up. (page 9 line 166)

Reviewer #3: The greater frequency of subclinical RHD among siblings may reflect the effect of common exposures rather than genetic influences. Therefore, the authors may wish to place less emphasis on the reasons for this finding, and instead discuss the practical implications for screening programs in greater detail.

This is an important point and we have therefore re-written this section of the discussion trying to enlarge on the implications (page 9 line 179)

--------------------

Editorial and Data Presentation Modifications? Use this section for editorial suggestions as well as relatively minor modifications of existing data that would enhance clarity. If the only modifications needed are minor and/or editorial, you may wish to recommend “Minor Revision” or “Accept”.

Reviewer #1: (No Response)

Reviewer #2: Minor modifications are mentioned in above sections.

Reviewer #3: (No Response)

--------------------

**Summary and General Comments** Use this section to provide overall comments, discuss strengths/weaknesses of the study, novelty, significance, general execution and scholarship. You may also include additional comments for the author, including concerns about dual publication, research ethics, or publication ethics. If requesting major revision, please articulate the new experiments that are needed.

Reviewer #1: In this study, the authors describe a study that followed up children diagnosed with latent RHD in 2017.

After 57 months of follow up, two thirds of 36 patients who they were initially identified as definite still remained in the definite category, while a third regressed and some had started developing symptoms of clinical disease.

Comments

While it is impressive that the authors were able to track the study participants five years after the initial screening, there are several issues to address:

What was the rationale for the follow up? Was it a natural history study? What was the sample size estimation and did it account for the changes in valve morphology at study end? Were these participants followed up between year 1 and year 5, were there any strategies for surveillance for worsening including recurrences of acute rheumatic fever? How was equipoise regarding BPG administration to latent RHD participants handled?

This study reflects a general approach to latent RHD screening that was done 2010 and 2015. Typically, cross sectional screening for latent RHD identified children with various grades of latent RHD according to the 2012 WHF and were then followed up. Most of the earlier similar studies have been cited by the authors.

The earlier studies, including this one, had several limitations including lack of follow up to detect episodes of acute rheumatic fever recurrence that could have led to progression of the valve lesion, lack of standardised administration of benzathine penicillin, no proper sample size estimation to allow for advanced statistics to objectively measure the stated outcomes such as progression and regression of valve lesions. Consequently, these designs have been over taken by more recent studies with better planned sample size and follow up plan that allow for proper estimation of outcomes including changes in valve lesion morphology. Secondly, until we have evidence on the role of BPG on latent RHD outcomes, blinded screening and follow up may not be useful as this will generate information already known from the natural history of RF/RHD.

The original study (started in 2012/3) was set up to determine the prevalence of latent RHD in this rural area of Ethiopia which is typical of much of rural sub-Saharan Africa and as mentioned above the population sample size (n=987) was chosen so as to give a prevalence estimate with a standard error of 1.0%. This study size therefore determines the numbers available for follow-up. Because of the paucity of data on RHD in rural African populations and the interest that this study created, we carried out the follow up study with the primary aim of studying the natural history of the disease and determining the feasibility of carrying out long-term studies in such populations.

Although the type of study outlined is desirable, it would be very difficult to carry out in rural populations such as we describe. The health service in this area is represented by health posts or health centers which provide a very limited service. The specialist hospital is inaccessible for most and therefore surveillance and the identification of recurrences of rheumatic fever were not possible although we asked about clinical symptoms of RHF in the questionnaires. For fairness (and as agreed by the local research ethics committee) we asked all definite and borderline cases of RHD to take BPG although, as we describe, only two of the cases actually took BPG.

Reviewer #2: It is one of several papers available on follow up of subclinical or latent RHD. However authors have in addition studied the family members of both RHD cases and controls.

Reviewer #3: (No Response)
